# Next-Generation Sequencing Applications for Inherited Retinal Diseases

**DOI:** 10.3390/ijms22115684

**Published:** 2021-05-26

**Authors:** Adrian Dockery, Laura Whelan, Pete Humphries, G. Jane Farrar

**Affiliations:** The School of Genetics & Microbiology, Trinity College Dublin, Dublin 2, Ireland; whelanl1@tcd.ie (L.W.); pete.humphries@tcd.ie (P.H.); jane.farrar@tcd.ie (G.J.F.)

**Keywords:** genetic diagnosis, inherited retinal disease, rare disease, retina, sequencing, diagnostics, macula, genomics, variant interpretation, eye

## Abstract

Inherited retinal diseases (IRDs) represent a collection of phenotypically and genetically diverse conditions. IRDs phenotype(s) can be isolated to the eye or can involve multiple tissues. These conditions are associated with diverse forms of inheritance, and variants within the same gene often can be associated with multiple distinct phenotypes. Such aspects of the IRDs highlight the difficulty met when establishing a genetic diagnosis in patients. Here we provide an overview of cutting-edge next-generation sequencing techniques and strategies currently in use to maximise the effectivity of IRD gene screening. These techniques have helped researchers globally to find elusive causes of IRDs, including copy number variants, structural variants, new IRD genes and deep intronic variants, among others. Resolving a genetic diagnosis with thorough testing enables a more accurate diagnosis and more informed prognosis and should also provide information on inheritance patterns which may be of particular interest to patients of a child-bearing age. Given that IRDs are heritable conditions, genetic counselling may be offered to help inform family planning, carrier testing and prenatal screening. Additionally, a verified genetic diagnosis may enable access to appropriate clinical trials or approved medications that may be available for the condition.

## 1. Introduction

A primary focus in ocular genetics globally is accurate genotyping of patients with rare inherited retinal diseases (IRDs). Next-generation sequencing (NGS) has been a common strategy employed in many countries to achieve this goal for several years [1,2,3,4,5,6,7,8,9,10,11,12,13,14,15,16]. This review focuses on the various methods and strategies that are being implemented to elucidate the genetic pathogenesis of IRDs and provides an overview of how these approaches have evolved. IRDs have an estimated prevalence of 1 in 4000 [17]. With a current global population of approximately 7.8 billion [18], it is estimated that approximately 2 million people currently have some form of IRD.

As a global community involved in ocular genetics, the common goal is to achieve a diagnostic success rate of 100% for all IRD patients enrolled in clinical studies. This objective, however, presents several challenges. Firstly, over 270 genes have been associated with the aetiologies of IRDs (RetNet, Retinal Information Network, https://sph.uth.edu/retnet/ accessed on 20 April 2021) [19]. Furthermore, extensive diversity in clinical presentation due to mutations even within a single IRD gene, as well as intersecting clinical phenotypes and phenocopies, is encountered. Mutations in disease genes may affect the retina in isolation, or may have more systemic effects. For example, there are 80 systemic conditions with a retinal phenotype and 200 genes that not only affect retinal health but also the central nervous system, kidneys or heart [20]. Such complexity makes it near-impossible for a diagnosis to be achieved in most instances solely on the basis of disease phenotype [2,21,22,23]. Furthermore, even a single pathogenic variant can manifest with phenotypic variability [24]. For some IRDs, modifier loci have been identified, somewhat blurring the borders between Mendelian and polygenic forms of IRD and mirroring similar observations with other disease aetiologies [25,26].

In this review, we aim to provide an overview of the NGS strategies employed globally to maximise the detection of IRD-causing mutations. This includes the use of targeted gene panels for all IRD phenotypes or phenotypic subsets; whole-exome sequencing (WES); whole-gene sequencing, whereby an IRD gene’s 5′ and 3′ sequences, exons and introns are interrogated; whole-genome sequencing (WGS); and bespoke methods to compliment other strategies, such as structural variant (SV) detection and copy number variant detection (CNV), among many others. In parallel with the use of the above technologies for the identification of candidate IRD-causing sequence variants, a wide array of methods to explore the functional effects of sequence variants have also been developed, and these are discussed.

While NGS technologies have enabled rapid characterisation of the genetic architecture of IRDs in many disease cohorts with diagnostic rates often approximating 70% [27], much still remains to be optimised. Strategies currently under development to further improve diagnostic rates are reviewed herein, as are the approaches being employed to enable interpretation of novel coding and non-coding candidate variants. Additionally, given the availability of increased numbers of WGS sequences from IRD and control populations, a greater focus is placed on the elucidation of the genetic modifier loci that may influence the effect(s) of the primary disease-causing mutations. An overview of the findings to date is provided.

## 2. IRDs—Target Panels and Whole Exome Studies

To elucidate the genetic contributions to IRDs, DNA, typically isolated from saliva or peripheral blood sample, is analysed. Optimal processing of the sample will depend on which form NGS is to be employed. In terms of both cost and data generated, NGS methods ranging from low to high involve targeted sequencing (TS), whole-exome sequencing (WES) or whole-genome sequencing (WGS). WES exclusively captures the protein-coding exons, but only accounts for approximately 1% of the genome. It is important to note that exon-based sequencing is likely to also reliably detect intronic variants located close to the targeted exons, such as non-canonical splice site variants, which are known causes of IRDs [28,29,30]. WGS is significantly more comprehensive including introns, promoters, and intergenic regions; in principle sequencing every nucleotide possible in a sample. TS typically captures the smallest amount of genetic information but does so in a completely customisable manner. For example, some IRD phenotypes are associated with pathogenic variants in a very small number of genes, but some of those genes may also be known to harbour pathogenic deep-intronic variants. In this case, adopting a TS approach would be more fruitful than WES. Arguably, WGS could also be used for this purpose but would generate more off-target data requiring significantly greater levels of analysis and storage and the on-target data would likely be less than a TS approach.

The benefits of TS are that it is an economical method of focusing sequencing capacity in smaller genomic regions including noncoding regions, therefore maximizing the coverage of clinically relevant genes. Enhanced coverage translates to greater sequencing read depth which is valuable, for example, to increase the resolution of detecting genetic variants and to detect smaller levels of heteroplasmy in the mitochondrial genome, or mosaicism in the nuclear genome [31,32,33,34]. By reducing the size of the region of genome sequenced per sample, a greater number of samples can be multiplexed together and processed in the same sequencing run. There are other cost-savings elements to TS, smaller file sizes allow for cheaper storage and faster processing. Moreover, targeting specific regions of the genome previously implicated in IRDs, can massively reduce the risk of detecting secondary or incidental findings.

For these reasons, TS strategies have frequently been employed for IRD screening for many years. Shortcomings of a TS strategy are that it often involves multiple gene panels for different conditions and if new IRD genes are identified or new variant associations are made for genes outside of the panel, a panel redesign will be required to include them. It is possible to use TS to detect indicators of large structural variants; however, such genomic breakpoints would likely have to occur within the captured loci reducing the likelihood of identification [1].

The customisable element of TS has become increasingly valuable with the recent detection of several population-enriched rare pathogenic variants likely due to founder effects. For example, a novel *PDE6B* variant was observed in the Māori IRD participant group and is likely to account for 16% of all recessive IRDs in that population [35]. Similarly, *EYS* gene variants were found to be causative in 51% of a RP cohort from Japan [36]. This discovery is not unique, as several other parallel studies have revealed similar founder mutations in their target populations, for example, Belgium, *RAX2* [37]; Costa Rica, *RPE65* [38]; Finland, *CERKL* [39]; Japan, *EYS* [36]; Spain, *RP1* [40] and *ABCA4* [41]; Jewish community in Caucasia, *PDE6B* [42]; Pakistan, *ABCA4* and *NMNAT1* [43]; Guyana, *BBS9* [44]; and Faroe Islands, *MERTK* [45]. The enrichment of these variants, several of which are large structural variants, emphasises the value of population-specific TS panels to target and detect mutations and mutational breakpoints that may be missed by commercial generic gene panel sequencing or even WES.

The use of WES has increased in popularity in recent years (Table 1) compared to previous metadata reported [27] and has many advantages over the TS approach. An effective TS panel design can be optimised with prior knowledge of the spectrum of mutations capable of causing the patient’s condition. This includes but is not limited to, knowledge of possible founder mutations in the population, all possible genotype–phenotype associations and breakpoint locations of any large structural variants that may exist. WES is agnostic to these issues. Although WES is not capable of detecting deep intronic mutations without modifying the method, it enables exonic variants to be detected even if their relevance is not entirely elucidated at the time of capture. This provides the potential for future interrogation of WES data as new IRD genes are discovered. Importantly, WES allows for the potential future resolution of a previously unsolved diagnosis.

Further to this point, many disease phenotype-based gene panels are very specific and therefore typically target only a small number of genes, not allowing for the possibility of new genotype–phenotype correlations or ambiguous phenotypes. Indeed, it was recently established that 23% of cases analysed would not have been resolved if they were sequenced by a commercial panel designed specifically for a patient’s phenotype [81]. In the same study, it was also found that for 26% of participants, the cheapest applicable commercial gene panel would have been costlier than performing WES for those patients.

Several large IRD screening studies in recent years have sought to identify the genes responsible for the largest proportions of their cohorts’ IRDs. In the UK, over 3000 pedigrees were reviewed, and it was determined that 135 IRD genes contributed to the genetic pathogenesis of the cohort. Interestingly, 70% of resolved cases were deemed to have causative mutations in 20 genes only [82]. Similarly, in over 5000 pedigrees with genetic eye conditions from Canada and the US, 68% of pathogenic or likely pathogenic mutations were identified in just 10 genes [78]. Both of these large studies identified *ABCA4*, *USH2A* and *RPGR* as the top three genes contributing to IRDs.

Although clearly not 100% effective, smaller whole-gene panels may be very effective as a first-tier screening approach. Several gene associations have been recently flagged as unlikely to be as pathogenic as initially reported. Nineteen percent of queried autosomal dominant retinitis pigmentosa (adRP) genes were deemed to harbour variants unlikely to be disease-causing for reasons relating to their respective allele frequencies or variant interpretation at that time [83]. Such variant “false positives” are shortcomings of the diagnostic odyssey, and this implies that, for an initial screening procedure, there may not be the need to screen as many of the genes and variants that are typically included in large gene panel screening studies.

It is important to also note that there have been many reports of the occurrence of multiple IRDs within the same family, or even within a single individual. Although individually IRDs are rare globally, the concurrence of multiple IRDs in a patient or pedigree unfortunately represents another diagnostic challenge. Our team has previously reported a pedigree in which five affected members of the family were broadly categorised as RP phenotypes. After genetic investigation it was revealed that four of these individuals were homozygous for a *FLVCR1* variant, while the remaining affected patient was compound heterozygous for pathogenic variants in *NR2E3* [84]. Similarly, in a US study, involving three IRD pedigrees, each given an initial diagnosis of RP, one with a dominant RP and the other two with a dominant, incompletely penetrant RP, it was found that multiple IRD genes were responsible for various affected individuals in each of the three families: both *USH2A* and *RP1* was segregating in one family; *PRPH2* and *CRX* in a second family and *PRPH2*, *PRPH8* and *USH2A* in the third family [85]. These studies, however, are trumped in complexity by Birtel et al.’s analysis of a single family with four different IRDs each caused by distinct pathogenic variants and inheritance patterns: father, *RHO*, dominant RP; mother, *ABCA4* and *CACNA1F*, recessive Stargardt and CSNB; first son, *CACNA1F*, CSNB; second son, *MITF*, dominant Waardenburg syndrome [86]. These are some examples of the many that exist, illustrating the complexity of IRD screening and reinforcing the necessity of thorough clinical and genetic investigation prior to genetic counselling [87].

## 3. Expanding IRD Diagnosis via Whole-Gene or WGS

For any laboratory electing to use NGS, it is essential that the limitations of the NGS approach to be employed are known. Failure to appropriately sequence the target genomic sites clearly will limit the success rate from the outset. Three consistent biases that exist for WES but not WGS are strand bias, evenness of coverage and the proportion of transcripts covered in their entirety. Interestingly, it has also been found that WGS provides a 3% better coverage of exons compared to WES, 98% versus 95% [88]. In another study it was found that the WGS approach offered superior detection of structural variants, variants in regulatory regions and detection of variants in GC-rich regions compared to WES [74]. However, this additional superior detection comes at a significant financial cost. A review of studies that used WES and WGS for clinical practice revealed that the price range for WGS studies was approximately five times higher for WGS on a per sample basis [89].

Costs incurred by WGS include not only the upfront cost of sequencing, but also additional downstream expenses. WGS produces vastly more data, thus immediately requiring additional computational power, people hours and storage to process. Although storage issues may be a limiting factor in the budgets of most research groups, policies regarding data storage can be readily adjusted to meet the needs of the research group, as the needs of two research groups will rarely be the same. This bespoke approach is advisable to avoid issues such as inadequate infrastructure and overspending. Raw sequencing files (such as .fastq files) and output files (variant call files, .vcf) are relatively small in comparison to the alignment files (such as .bam files) that need to be produced as part of the analyses [90,91,92]. Given this information, it is possible to reduce the capacity required for long term storage of sequencing data by electing to discard the alignment files but keeping the input and output files so that the analysis can be repeated at a later date and outputs can be compared for discrepancies and newer discoveries. However, increased storage will be required again upon reanalysis, as new alignment files will be created as part of the process.

Another viable alternative in reducing the disk footprint of alignment files, is the use of additionally compressed formats, such as .cram files. This format uses reference-based compression, only storing base calls that differ to the reference genome used. This compression can be either lossless or can incur a reduction in base quality scores corresponding to the level of compression. Even the lossless format offers a 40–50% reduction in space required by comparison with BAM [93]. In addition, the use of WGS as a second-tier approach, for cases that remain genetically unresolved following first-tier sequencing will decrease data-storage demands. More research groups are now making the move to cloud-based storage for their NGS data and minimising the amount of data stored has a direct impact on cost [94]. It is important to note that sample processing and analyses are available via cloud-based solutions also, and may be an attractive option for research groups lacking the necessary in-house infrastructure to process NGS data [95].

The additional cost of larger-scale analysis is not the only hazard associated with this data management. Both WES and WGS have an increased likelihood of carrying intrinsic responsibilities regarding the management of incidental or secondary findings (SFs) unrelated to the initial indication for sequencing. For example, Hart et al. (2018) found that a SF is detected in 1.7% of patients who undergo WES [96]. Some IRD studies have employed a nested targeted approach, wherein the entire genome of an individual is sequenced but only variants in genes relevant to the IRD phenotype are interrogated by use of variant filtering with a virtual gene panel. This still provides benefits over traditional targeted panels, as it also includes sequencing of non-coding regions, as well as the potential for analysis of an expanded panel in the future. For example, Carss et al. (2017) performed WES on 117 individuals, identifying pathogenic variants in 59 cases [74]. Forty-five of the unresolved cases then underwent WGS and positive candidate variants were identified in an additional 14 cases. This approach is likely chosen due to the immense volume of data produced by WGS and WES, and the need to more rapidly analyse the most relevant data available.

This approach also limits the possibility of detecting SFs. In SF v1.0, The American College of Medical Genetics and Genomics (ACMG) recommended analysis of 56 medically actionable gene–phenotype pairs which was then updated to a panel of 59 genes in v2.0 [97,98]. ACMG SF v3.0, recommending the analysis of SFs in 73 gene–phenotype pairs, was very recently released [99,100]. Of particular interest to the ocular genetics community, ACMG SF v3.0 now includes the *RPE65* gene. The *RPE65* gene was included on the basis that an FDA-approved gene therapy now exists for biallelic RPE65-retinopathies and that patients may derive additional benefit from earlier detection and therapeutic intervention. The ACMG recommends application of these SF guidelines in a clinical setting as opposed to a research setting. Nonetheless, as with all genomic testing, it is imperative that the patient’s interests are at the forefront. ACMG currently recommend that patients/guardians have the choice to opt-out of SF testing. This highlights the necessity of an appropriate and comprehensive pre-testing consent procedure. This includes and is not limited to information pertaining to what will not be disclosed should the patient/guardian chose to abstain from SF analysis and thorough pre-test and post-test counselling.

Despite additional costs, there may be diagnostic benefits to employing WGS to resolve genotypes. Lionel et al. investigated 103 cases of diverse genetic disorders comparing WGS to targeted panel sequencing. Not only was the solve rate superior when WGS was used, 41% versus 24%, 18 diagnoses were made based on structural variants or intronic variants that were not captured by the TS method [101]. Regardless of the substantial number of genes identified and targeted by TS, as estimated from studies to date, the genetic cause of 43% of all IRDs patients remains unknown and suggests the need for more studies to employ WGS (Table 1). These missing genetic aberrations may reside in introns or intergenic regions, both of which are captured by WGS. There is also the possibly of novel IRD gene discovery that is facilitated by WGS. The superior uniformity of genome coverage enabled by WGS also allows for greater sensitivity when detecting copy number variants (CNVs) that are notoriously difficult to detect by TS and WES.

A cost-effective alternative that retains many of the same benefits as WGS is whole-gene sequencing (GS). GS enables the capture of exonic, intronic and 5′ and 3′ regulatory regions for a target gene of interest but has many of the same limitations as the TS approach, including strand bias and GC-rich impedance to capture. GS has been utilised very successfully for cohorts with phenotypes associated with monogenic or near-monogenic causes. For example, individuals affected with incomplete congenital stationary night blindness (icCSNB) present with a recognisable phenotype. This form of icCSNB is primarily associated with mutations in the *CACNA1F* gene. In a recent large genotyping study of icCSNB–CACNA1F patients (*n* = 189), 4% of *CACNA1F* causative variants were attributed to intronic and synonymous mutations [102]. It is also probable that there are additional intronic variants yet to be designated as pathogenic in the unresolved portion of this cohort.

Similarly, Khan et al. investigated 1054 unresolved Stargardt cases with a GS approach. Stargardt disease is predominately caused by biallelic variants in the *ABCA4* gene. The authors of the study used a single-molecule molecular inversion probes (smMIPs) approach, which proved reliable and cost-effective. Their study revealed the presence of pathogenic SVs and deep-intronic variants in 25% of biallelic cases [103]. The smMIPs method is gaining in popularity given its superior target capture and low cost compared to other TS capture methods. In a recent comparative study, 176 IRD patients were analysed with both smMIPs and TS. The smMIPs approach demonstrated enhanced target coverage (97.3% versus 93.9%) and was five times more cost effective when greater than 500 samples were analysed [104].

The GS approach has also been combined with probes for other IRD genes to investigate if this combinatorial strategy could significantly improve diagnostic rates for a range of IRDs when compared to traditional exon-based TS IRD panels. The study design encompassed a second-tier approach for patients who had one previous variant found in *USH2A*, *ABCA4* and *CEP290.* These whole genes, plus exons of 76 additional IRD genes and pathogenic intronic regions of two IRD genes were sequenced in an effort to resolve the “one-hit” patients. An overall diagnostic rate of 58.6% was achieved; two copy number variants were detected in *USH2A* [105]. Although this diagnostic rate was no higher than the average study (Table 1), it does represent significant improvements that can be made to address the large proportion of unresolved patients identified by standard screening studies. The structural variants established in this study would likely not have been detected by use of a more traditional, purely exon-targeting design.

An improved GS study design as outlined above may have additional advantages. The *RPGR* gene is one example of an IRD gene that includes regions that are challenging to sequence comprehensively with traditional TS or WES; sequencing through ORF15 is impeded due to a low-complexity sequence composition [106]. However, it is vital to capture this gene as, for example, it accounts for nearly 40% of X-linked retinopathies in the UK. This makes pathogenic variants in this gene the third most-prevalent cause of IRDs in this population [82]. In an Italian cohort of 48 *RPGR*-related RP cases, approximately half had mutations in ORF15 and presented with a more severe phenotype than the other causative variants in exons 1–14 of *RPGR* [107]. It has been suggested that the sequence coverage of ORF15 could be optimised by modifying NGS library preparation, reducing false-negatives, miscalled variants and false-positives when compared to traditional methods [108]. For this reason, many recent NGS screening studies have adopted bespoke approaches to sequencing *RPGR*, including entirely separate analyses or spiking the NGS libraries with separately generated amplicons for *RPGR* [50,53,56]. In another study improved alignment of sequencing reads mapping to the ORF15 region by using a de novo assembly approach were reported. The accuracy of sequencing can be quickly determined for males when analysing the RPGR gene, as variants called in error will likely not be represented in every sequencing read mapping to this region. Therefore, heterozygous variant calls can be readily identified as errors, since males have only one copy of RPGR. This de novo assembly approach reduced the number of false-heterozygous calls in males and improved the accuracy of indel calls [109].

Another example of genes that benefit from tailored GS design are those encoding the opsins, *OPN1LW* (red cone cells) and *OPN1MW* (green cone cells). These genes encode photopigments in the retina and pose an interesting challenge to sequencing efforts. These two genes are 96% homologous which introduces unique challenges for the IRD gene panel, as short-read sequencing may be unable to determine the best alignment option when mapping back to the genome [110]. A new two-step method from Atilano et al. has demonstrated that long-range PCR can generate specific long amplicons that can be more readily mapped back to the genome [111]. This approach offers a solution that can be analysed separately, by direct sequencing of the amplicons, or alternatively, as part of a larger panel if long-read sequencing (LRseq) is used.

Sequencing the entirety of a gene also facilitates the detection of variants in the upstream regulatory regions which have been implicated in retinal disease previously, for example in Blue-Cone Monochromacy (BCM) [112]. In this condition, a c. −71A>C promoter mutation was initially thought to decrease expression and cause a deutan colour vision deficiency. However, after functional analyses, the mutation was revealed to result in more than double the wild-type expression level of the gene [113]. Other deletions in this area have also been shown to result in BCM phenotypes, suggesting that this gene is sensitive to alterations in both under and overexpression [114,115]. Similarly, Radziwon et al. used luciferase reporter assays to assess upstream variants detected in the *CHM* gene in patients with choroideremia. Both probands had variants at position c. −98: C>A and C>T. Both mutations led to a reduction in luciferase activity and furthermore, the promoter region for *CHM* was defined as the region encompassing nucleotides c. −119 to c. −76 [116].

Regulatory mutations are often difficult to interpret, particularly for genes associated with recessive forms of inheritance. Previously, consanguineous pedigrees have been useful for identifying homozygous variants in these cases, such as *NMNAT*-related Leber congenital amaurosis (LCA) [117]. Variant interpretation can be further complicated as such variants may not have strong effects on gene expression. In a recent study of promoter variants in *ELOVL4* two variants were found, c. −236 C>T (rs240307) and c. −90 G>C (rs62407622) which resulted in 18% and 14% reduction in expressivity, respectively. However, as the patient in question had the variants in *trans*, a severe phenotype was observed, much more than would have been expected from the modest effect of the two variants analysed separately [118]. This detrimental synergistic effect may emphasise the threshold sensitivity of retinal tissues and cell components to the dosage levels of this protein and its downstream effects.

## 4. Copy Number and Structural Variants

As discussed above, TS and WES methods are the most universally utilised, yet they are largely incapable of detecting large copy number variants (CNVs), structural variants (SVs) and chromosomal rearrangements. In 2018, an extensive literature-mining endeavour revealed that 1345 copy-number variants (CNVs)—specifically, 317 unique variants—had been reported in 81 distinct IRD genes. When further analysed, the size of the gene correlated with the reported numbers of CNVs associated with that gene. Additionally, many of these large variants affected non-coding and potential *cis*-regulatory elements [119]. The relevance of such variant types is now recognised, and guidelines have been published to assist in the interpretation and classification of them, similarly to those published in recent years for single-nucleotide variants [120,121].

CNVs and SVs can also vary significantly in the complexity of their rearrangements. Gross deletions have previously been detected in many genes, including *BEST1*, *EYS*, *MERTK*, *USH2A* and many more from the aforementioned study alone [119]. Large deletions have also been reported in *RPGR* [122], *CHM* [58], *OPN1LW*/*OPN1MW* [123] and *USH1C* [1], to name but a few. Deletions are likely to be the most detectable CNV type given that most studies employ WES or TS to detect mutations. Homozygous deletions are the most readily detectable from using these methods as the read coverage over the deleted region would be zero, given no template exists for capture or amplification. Heterozygous deletions may be under-reported when using WES or TS, if significant amplification has occurred, which may unintentionally normalise the ultimate read depth aligned to the deleted region. For similar reasons, duplications can be very difficult to detect with these methods. However, such mutations can be more readily detected by WGS due to the superior and more even coverage, or by more specific approaches, such as targeted locus amplification [119]. Some regions of the genome, such as the RP17 locus, have been shown to harbour many complex CNV and SV variants associated with IRDs. These convoluted rearrangements resulted in the interference of the surrounding genome architecture, disrupting enhancer–promoter interactions, and resulting in aberrant gene expression [124].

Genomic rearrangements are more likely to be detected by the presence of broken sequencing reads when aligned back to the reference genome. This occurs when a read, or pair of reads, partially align to one part of the genome and partially to somewhere quite different. This is applicable to translocations and inversions, as unlike CNVs, the read depth is not expected to be altered in these scenarios. Given the significant presence of retrotransposon sequence in the human genome, it is not surprising that several retrotransposons insertions have been reported to disrupt the functionality of IRD genes [125,126,127]. The *BBS1* gene in particular, has been recently reported to harbour retrotransposons causative of disease [128,129]. Retrotransposons, much like other large genomic insertions, can be difficult to detect as they are unlikely to disrupt read depth in the genomic region to which they have relocated to, since alignment tools will align these reads to their original positions in the genome. Broken reads may indicate that a rearrangement has occurred. If breakpoints are detected, the region can be directly sequenced to shed light on the nature of the SV. Alternatively, a de novo assembly approach may be used to reconstruct the queried genome [130]. This approach will likely only be beneficial in the case of WGS, since TS or WES will likely not have sequenced the insert because the original genomic region was not an intended target.

In one study, involving an investigation of *PRPF31*-related disease, 45% of probands (10 of 22) tested positive for a CNV. The *PRPF31* gene has no obvious sequence elements that may make it particularly susceptible to genomic rearrangement, such as long interspersed nuclear elements (LINE) and long terminal repeat (LTR) elements [131]. The study emphasises the importance of integrating CNV and SV detection into screening protocols, even for genes that may not appear to be conventionally susceptible to genomic rearrangements. The estimated prevalence of causative SVs in IRD cases is roughly 10% [51,131,132,133]. This is similar to findings from other rare disease cohorts as 12% of developmental disorders are estimated to be caused by pathogenic CNVs, therefore CNV and SV detection is recommended to be incorporated into first-tier testing for that set of conditions [134]. In a large hearing loss screening study of over 1000 patients, 18% of resolved patients were found to have causative CNVs [135]. CNV detection has also proven very useful in diagnosing atypical syndromic IRD cases resulting in novel genotype–phenotype associations and the refinement of complex phenotypes in multiple cases [136].

Many of the NGS methods discussed so far have revolved around short-read sequencing; however, long-read sequencing is arguably the superior approach for detection of SVs and CNVs. Short-read sequencing is generally preferred to ensure that high-quality data are produced [137]. However, this technology is greatly hindered by features of the genome, such as repetitive elements, which are not only abundant in our genomes, but also known to increase the likelihood of an SV event occurring in IRD genes [123,138]. Long-read sequencing offers superior sequencing of such regions and offers a chance to more accurately recapitulate patients’ true genomic sequences through the use of de novo assembly [139,140,141]. Results from several studies to date have revealed IRD-causing SVs by the use of long-read sequencing, and in some cases, concluded that the complexity of the SV was such that it was likely not possible to fully resolve it by short-read sequencing [44,142].

Another useful application of long-read sequencing is determining the phase of potentially causative recessive variants. Determining the phase of variants is of critical importance, as it may determine whether causative variants have been established, if in *trans*, or not, if in *cis*. This task is challenging for IRD cases primarily for two reasons. Firstly, variants causative of Mendelian IRDs are extremely rare in most cases. This prevents the establishment of known haplotypes or complex alleles in most cases that may otherwise indicate that the two detected variants are likely in *cis*. Secondly, many IRD screening endeavours are still in their infancy. This results in the widest possible age range of patients, since even patients with paediatric onset of their condition, may be elderly when screened. This can often make segregation analysis difficult, as many of their close family members may be immobile or deceased. Long-read sequencing offers the interpreter a greater chance of capturing both variants of interest within the same sequencing molecule and therefore determining phase of the variants without the need for additional family members [140].

## 5. Modifiers of IRD Phenotypes

There are multiple examples of phenotypes manifesting significantly differently in IRD patients, even when harbouring the same mutation [24]. Such variability may be in overall severity, age of onset or pattern of degeneration. While some instances may be partially explained by variant haplotypes or known interactions with other genes, much of the source of variation remains unknown [143]. It has also yet to be established how much of this unknown contribution is genetic.

Pathogenic variants in *PRPF31* are the second most common cause of autosomal dominant retinitis pigmentosa [144]. PRPF31 is a universally expressed splicing-factor and has a vital role in the processing of pre-mRNA. However, reduced expression of this ubiquitous splicing-factor results in isolated retinal restricted disease, retinitis pigmentosa. It has been shown in multiple studies that reduced levels of PRPF31 in the retina results in the mis-splicing and reduced expression of several key IRD genes associated with phototransduction and RNA processing [145,146]. This indirect mechanism of action may partially explain the incomplete penetrance and variable severity frequently reported with this IRD gene [144]. It has also been shown that minisatellite repeat elements (MSR1) proximal to the promoter of *PRPF31* can modify the expression of the gene. Alleles with four copies of the MSR1 were shown to correlate with asymptomatic individuals, while alleles with three copies of MSR1 greatly reduced the expression of *PRPF31* [147].

Another key factor in the manifestation of an IRD relates to the naturally occurring expression levels of the implicated genes. In a study by Green et al., IRD genes typically associated with incomplete inheritance significantly correlated with greater variability in levels of expression in a healthy population in several tissue types, including the eye. This implies that *cis* and or *trans* elements may be influencing variability in expression and in turn, contribute to disease states in patients [148]. This may also result in higher-than-expected allele frequencies for pathogenic variants located in these genes in “healthy” control databases, such as gnomAD [149]. 

Several IRD genes have been found to harbour pathogenic splice mutations. Splice mutations are associated with variable severity as the percentage of wild-type transcript that can still be produced is dependent on the specific mutation in question [150]. It is therefore plausible that some variants may only have marginal disruptive effects on normal splicing and result in a sub-clinical phenotype. It is important that each of these variants is evaluated to determine aberrant effect(s). They can be assessed by a variety of means. The most readily available method to assess variants involves use of plasmid transfection to recapitulate the patient’s mutation in mammalian cells. Minigene and Midigene constructs incorporating exons and introns for the IRD gene and including the candidate splice mutation have been hugely successful in providing empirical evidence of the functional effect of candidate splice variants [150,151,152,153]. Patient-derived cell and/or 3D retinal organoid models while more laborious to create, remove the need for transient expression of mutations as the mutation is already present in the patient’s cells. Organoids provide a more retina-like simulated environment to more accurately evaluate splice processing and thus have been utilised for the interpretation of multiple IRD variants [154,155,156]. 

The *RPGR* gene is another example of an IRD gene associated with variable disease severity and age of onset. In the case of *RPGR*, this is particularly true of female carriers of pathogenic variants. Random X-inactivation plays a decisive role on the severity of the IRD manifestation. In a recent analysis, blood and saliva samples were taken from 77 female carriers from 41 RPGR–IRD pedigrees. These samples were analysed for their methylation patterns. It was found that X-inactivation ratios correlated with clinical severity and could be useful indicators for prognostic purposes [157]. Another common feature of *RPGR* pathogenesis is that missense variants often lack an ability to interact with other key IRD gene products which results in the failure of RPGR to correctly localise to the cilia as required [158]. Interestingly, this means that polymorphic variation in the other interacting IRD genes, such as *IQCB1* and *RPGRIP1L*, can further modify the dynamics of this disrupted interaction [159].

Given that many IRDs involve progression over time, age is another modifier of disease. In several studies that have been mentioned previously, it was noted that diagnostic rates are higher in younger patients. In Germany, the mean age of participants with a positive molecular diagnosis was 39.8 years of age, whereas it was 46.3 years of age for unresolved patients [55]. In the UK, patients enrolled with late-onset macular disease (≥50 years of age) were less likely to receive a genetic diagnosis (18.0%) compared with patients with an onset of less than 50 years of age (24.2%) [73]. In the US, Stone et al. also noted that younger entrants to their screening study were more likely to have their disease-causing mutation identified [79]. These findings suggest that the genetic causes of early onset IRDs are better established and possibly that ageing increases susceptibility to polygenic, or perhaps non-genetic factors, making the older patient cohort more difficult to solve by current genetic screening approaches.

## 6. Impacts on IRD Diagnosis Outside of NGS Testing 

The surge of NGS studies addressing diagnostic challenges in IRDs in the last decade has also encouraged further developments in other areas related to IRD diagnosis. Massive improvements have been made in ophthalmological imaging, not just in resolution, but also in applications and options available to imaging technicians [160]. These techniques help refine phenotypes and can inform whether or not more advanced forms of imagery may be useful for particular patients [161]. Machine learning (ML) is a branch of artificial intelligence that focuses on the concept that systems can learn from training data, such as expert-reviewed fundus images of IRD patients, and thereby learn to identify these patterns independently. This involves expert curation to obtain the training dataset but has the advantage of removing variability due to being a single assessor once training is complete. For example, many studies employ ML to assess fundus and optical coherence tomography images to train and predict outcomes for potential age-related macular degeneration (AMD) patients, including risk of progression to a more severe disease state and response to treatments [162,163,164,165,166,167,168,169]. This approach has enormous potential benefits for improvement of diagnostic and prognostic accuracy. For example, ML has been utilised for the detection of fleck lesions in fundus autofluorescence imaging, a characteristic feature of Stargardt disease. The ML approach could accurately identify and quantify fleck lesions, a potential outcome measure for future clinical trials [170]. Similarly, optic disc photography has been used to systematically detect abnormalities of the optic disc [171]. ML strategies may revolutionise the speed and accuracy at which new patients can be phenotyped in the clinic. More accurate phenotyping may have beneficial implications for NGS screening studies also, particularly those utilising phenotype-based gene panels. Improving the effectiveness of TS is likely to have the most widespread benefits given the relative low cost (less than $20 per sample), making TS the most readily accessible to diagnostic labs, especially those with constrictive budgets [79].

While significant advances in the clinical and genetic diagnosis of IRDs have been made, implementation of IRD screening is under resourced in many countries. A recent study into the cost-of-illness of IRDs in Ireland and the UK demonstrated that lack of access to genetic testing, the absence of an international patient registry and the issues regarding the reimbursement of therapies are common complexities faced by IRD patients in these countries [172]. In the UK, surveyed IRD patients noted that they appreciated pre-test counselling including a discussion of the possibility of an unexpected result [173]. Some countries are actively tackling the issue of integrating genetic services into future planning for their healthcare systems. Portugal and Iran are two of the latest countries to announce the launch of their nationwide registries for IRDs [174,175]. These registries aim to increase accessibility for individuals, while also providing a comprehensive dataset for investigators and clinicians to boost and develop their research. One such example is the French Plan for Genomic Medicine 2025 [176]. This plan aims to improve healthcare by organising and structuring new pathways to care and counselling while also reviewing the current challenges impeding the implementation of genomic testing in France. Despite the many great advances detailed in this review, the successful adaptation of IRD genomics services into national healthcare programs is rare [177]. Given that the technology and expertise is available and accessible to many research teams around the world (Table 1), combining the findings from these studies with effective national healthcare screening systems may enable better treatment and care through clinical genetic interventions for the patient [177].

## 7. Conclusions

We are now in the age of genomic medicine, where genetic evidence can often provide precise and robust diagnoses. This evidence may inform prognoses and may aid in directing care plans and therapeutic intervention. This growth has been supported by rapid enhancements in DNA sequencing methodologies, analysis tools and skillsets. The enormous impact of such advances is clearly exemplified in the field of IRDs. The true genetic complexity of IRDs is more fully appreciated with mutations in more than 300 genes implicated in IRDs among many other non-coding and modifying elements. It is clear that many challenges exist for genetic screening of IRDs, as evident from the approximate 40% portion of screened patients whose causative genetic elements are yet to be established. Many of these cases may be resolved in the future as more comprehensive techniques, such as WGS, are more routinely utilised. As such data becomes available, it will undoubtedly also increase our knowledge of genetic elements that have a modifying effect on IRD manifestations and severities. Genetic-screening diagnostic rates are likely to also benefit from advances in technologies related to clinical phenotyping that, where appropriate, may be augmented by machine-learning-based algorithms. Clearly another development that should enhance IRD screening endeavours is the development of national databases and genomics strategies to develop services and enhance clinical genetics collaborations nationally and internationally.

## Figures and Tables

**Table 1:** Screening studies of inherited retinal disease (IRD) populations. CRD = cone–rod dystrophy; LCA = Lebers congenital amaurosis; IRD = inherited retinal dystrophy; MD = macular dystrophy; RP = retinitis pigmentosa; TC = target capture; WES = whole-exome sequencing; WGS = whole-genome sequencing.

Country	Author	Year	Pedigrees	Solve Rate	Cohort Details	TC (Genes)	WES	WGS
Australia	Thompson [46]	2017	34	90	LCA	Yes	-	-
Brazil	Motta [47]	2018	559	72	IRD	Yes	Yes	-
China	Liu [48]	2020	800	60	RP	Yes	Yes	-
China	Gao [49]	2019	1243	72	RP	586	-	-
China	Liu [50]	2020	182	48	IRD	Yes	Yes	-
China (Han)	Huang [51]	2017	98	41	RP	-	Yes	-
China	Dan [52]	2020	76	57	IRD	Yes	Yes	-
China	Wang [53]	2018	319	39	IRD	Yes	Yes	-
Finland	Avela [54]	2019	53	77	IRD	Yes	-	-
Germany	Weisschuh [55]	2020	1785	69	IRD	Yes	-	-
Germany	Birtel [56]	2018	251	74	MD/CRD	Yes	-	-
Iran	Tayebi [57]	2019	50	72	IRD	Yes	-	-
Ireland	Whelan [58]	2020	710	70	IRD	Yes	-	-
Israel	Sharon [59]	2020	2420	56	IRD	Yes	Yes	Yes
Japan	Koyanagi [60]	2019	1204	30	RP	Yes	-	-
Japan	Numa [36]	2020	220	45	RP	Yes	-	Yes
Korea	Surl [61]	2020	50	78	LCA	Yes	Yes	-
Korea	Kim [62]	2019	86	44	IRD	Yes	-	-
Mexico	Zenteno [63]	2019	143	66	IRD	Yes	-	-
Norway	Holtan [17]	2020	650	32	IRD	Yes	-	-
Poland	Wawrocka [64]	2018	18	39	CRD	Yes	Yes	-
Polynesian and Māori	Vincent [35]	2017	16	44	IRD	Yes	-	-
Spain	Perea-Romero [41]	2021	3951	53	IRD	Yes	Yes	Yes
Spain	Martin-Merida [65]	2019	877	38	RP	Yes	-	-
Spain	Gonzàlez-Duarte [66]	2019	73	85	IRD	Yes	-	-
Spain	Diñeiro [67]	2020	100	45	IRD	Yes	-	-
Taiwan	Chen [68]	2020	60	53	IRD	Yes	-	-
Tunisia	Habibi [69]	2020	73	68	IRD	-	Yes	-
UAE	Khan [70]	2020	71	100	Pediatric IRD	Yes	Yes	-
UAE	Patel [71]	2018	75	82	IRD	Yes	Yes	-
UK	Jiman [72]	2020	106	49	Syndromic IRD	Yes	-	-
UK	Shah [73]	2020	655	43	IRD	Yes	-	-
UK	Carss [74]	2017	722	56	IRD	-	Yes	Yes
UK	Lenassi [75]	2020	201	64	Pediatric IRD	Yes	-	-
UK	Patel [76]	2019	277	25	Pediatric IRD	Yes	-	-
UK	Taylor [77]	2017	85	79	Pediatric IRD	Yes	-	-
USA and Canada	Goetz [78]	2020	5385	62	IRD	Yes	-	-
USA	Stone [79]	2017	1000	76	IRD	-	Yes	-
USA	Bryant [80]	2018	69	64	IRD	-	Yes	-

## Data Availability

No new data were created or analyzed in this study. Data sharing is not applicable to this article.

## References

[1] Dockery A., Stephenson K., Keegan D., Wynne N., Silvestri G., Humphries P., Kenna P.F., Carrigan M., Farrar G.J. (2017). Target 5000: Target Capture Sequencing for Inherited Retinal Degenerations. Genes.

[2] Bernardis I., Chiesi L., Tenedini E., Artuso L., Percesepe A., Artusi V., Simone M.L., Manfredini R., Camparini M., Rinaldi C. (2016). Unravelling the Complexity of Inherited Retinal Dystrophies Molecular Testing: Added Value of Targeted Next-Generation Sequencing. BioMed Res. Int..

[3] Consugar M.B., Navarro-Gomez D., Place E.M., Bujakowska K.M., Sousa M.E., Fonseca-Kelly Z.D., Taub D., Janessian M., Wang D.Y., Au E.D. (2015). Panel-based genetic diagnostic testing for inherited eye diseases is highly accurate and reproducible, and more sensitive for variant detection, than exome sequencing. Genet. Med..

[4] Ellingford J.M., Barton S., Bhaskar S., O’Sullivan J., Williams S., Lamb J., Panda B., Sergouniotis P., Gillespie R.L., Daiger S.P. (2016). Molecular findings from 537 individuals with inherited retinal disease. J. Med. Genet..

[5] Tiwari A., Bahr A., Bähr L., Fleischhauer J., Zinkernagel M.S., Winkler N., Barthelmes D., Berger L., Gerth-Kahlert C., Neidhardt J. (2016). Next generation sequencing based identification of disease-associated mutations in Swiss patients with retinal dystrophies. Sci. Rep..

[6] Riera M., Navarro R., Ruiz-Nogales S., Méndez P., Burés-Jelstrup A., Corcóstegui B., Pomares E. (2017). Whole exome sequencing using Ion Proton system enables reliable genetic diagnosis of inherited retinal dystrophies. Sci. Rep..

[7] Haer-Wigman L., Van Zelst-Stams W.A.G., Pfundt R., Born L.I.V.D., Klaver C.C.W., Verheij J.B.G.M., Hoyng C.B., Breuning M.H., Boon C.J.F., Kievit A.J. (2017). Diagnostic exome sequencing in 266 Dutch patients with visual impairment. Eur. J. Hum. Genet..

[8] Audo I., Bujakowska K.M., Léveillard T., Mohand-Saïd S., Lancelot M.-E., Germain A., Antonio A., Michiels C., Saraiva J.-P., Letexier M. (2012). Development and application of a next-generation-sequencing (NGS) approach to detect known and novel gene defects underlying retinal diseases. Orphanet J. Rare Dis..

[9] De Castro-Miro M., Tonda R., Escudero-Ferruz P., Andrés R., Mayor-Lorenzo A., Castro J., Ciccioli M., Hidalgo D.A., Rodríguez-Ezcurra J.J., Farrando J. (2016). Novel Candidate Genes and a Wide Spectrum of Structural and Point Mutations Responsible for Inherited Retinal Dystrophies Revealed by Exome Sequencing. PLoS ONE.

[10] Neveling K., Collin R.W., Gilissen C., van Huet R.A., Visser L., Kwint M.P., Gijsen S.J., Zonneveld M.N., Wieskamp N., de Ligt J. (2012). Next-generation genetic testing for retinitis pigmentosa. Hum. Mutat..

[11] O’Sullivan J., Mullaney B.G., Bhaskar S.S., Dickerson J.E., Hall G., O’Grady A., Webster A., Ramsden S.C., Black G.C. (2012). A paradigm shift in the delivery of services for diagnosis of inherited retinal disease. J. Med. Genet..

[12] Shanks M.E., Downes S.M., Copley R.R., Lise S., Broxholme J., Hudspith K.A., Kwasniewska A., Davies W.I., Hankins M.W., Packham E.R. (2012). Next-generation sequencing (NGS) as a diagnostic tool for retinal degeneration reveals a much higher detection rate in early-onset disease. Eur. J. Hum. Genet..

[13] Ge Z., Bowles K., Goetz K., Scholl H.P.N., Wang F., Wang X., Xu S., Wang K., Wang H., Chen R. (2015). NGS-based Molecular diagnosis of 105 eyeGENE^®^ probands with Retinitis Pigmentosa. Sci. Rep..

[14] Zhao L., Wang F., Wang H., Li Y., Alexander S., Wang K., Willoughby C., Zaneveld J.E., Jiang L., Soens Z.T. (2015). Next-generation sequencing-based molecular diagnosis of 82 retinitis pigmentosa probands from Northern Ireland. Qual. Life Res..

[15] Perez-Carro R., Corton M., Sánchez-Navarro I., Zurita O., Sanchez-Bolivar N., Sánchez-Alcudia R., Lelieveld S.H., Aller E., Lopez-Martinez M.A., López-Molina M.I. (2016). Panel-based NGS Reveals Novel Pathogenic Mutations in Autosomal Recessive Retinitis Pigmentosa. Sci. Rep..

[16] Weisschuh N., Mayer A.K., Strom T.M., Kohl S., Glöckle N., Schubach M., Andreasson S., Bernd A., Birch D.G., Hamel C.P. (2016). Mutation Detection in Patients with Retinal Dystrophies Using Targeted Next Generation Sequencing. PLoS ONE.

[17] Holtan J.P., Selmer K.K., Heimdal K.R., Bragadóttir R. (2019). Inherited retinal disease in Norway—A characterization of current clinical and genetic knowledge. Acta Ophthalmol..

[18] 2020 World Population Data Sheet Shows Older Populations Growing, Total Fertility Rates Declining—Population Reference Bureau. https://www.prb.org/2020-world-population-data-sheet/.

[19] RetNet Retinal Information Network. https://sph.uth.edu/RetNet/.

[20] Tatour Y., Ben-Yosef T. (2020). Syndromic Inherited Retinal Diseases: Genetic, Clinical and Diagnostic Aspects. Diagnostics.

[21] Carrigan M., Duignan E., Humphries P., Palfi A., Kenna P.F., Farrar G.J. (2015). A novel homozygous truncatingGNAT1mutation implicated in retinal degeneration. Br. J. Ophthalmol..

[22] Shankar S.P., Hughbanks-Wheaton D.K., Birch D.G., Sullivan L.S., Conneely K.N., Bowne S.J., Stone E.M., Daiger S.P. (2016). Autosomal Dominant Retinal Dystrophies Caused by a Founder Splice Site Mutation, c.828+3A>T, in PRPH2 and Protein Haplotypes in trans as Modifiers. Investig. Ophthalmol. Vis. Sci..

[23] Tee J.J.L., Smith A.J., Hardcastle A.J., Michaelides M. (2016). RPGR-associated retinopathy: Clinical features, molecular genetics, animal models and therapeutic options. Br. J. Ophthalmol..

[24] Zupan A., Fakin A., Battelino S., Jarc-Vidmar M., Hawlina M., Bonnet C., Petit C., Glavač D. (2019). Clinical and Haplotypic Variability of Slovenian USH2A Patients Homozygous for the c. 11864G>A Nonsense Mutation. Genes.

[25] Becker T., Pich A., Tamm S., Hedtfeld S., Ibrahim M., Altmüller J., Dalibor N., Toliat M.R., Janciauskiene S., Tümmler B. (2020). Genetic information from discordant sibling pairs points to ESRP2 as a candidate trans-acting regulator of the CF modifier gene SCNN1B. Sci. Rep..

[26] Bandres-Ciga S., Center T.A.G., Saez-Atienzar S., Kim J.J., Makarious M.B., Faghri F., Diez-Fairen M., Iwaki H., Leonard H., Botia J. (2020). Large-scale pathway specific polygenic risk and transcriptomic community network analysis identifies novel functional pathways in Parkinson disease. Acta Neuropathol..

[27] Farrar G.J., Carrigan M., Dockery A., Millington-Ward S., Palfi A., Chadderton N., Humphries M., Kiang A.S., Kenna P.F., Humphries P. (2017). Toward an elucidation of the molecular genetics of inherited retinal degenerations. Hum. Mol. Genet..

[28] Kortüm F., Kieninger S., Mazzola P., Kohl S., Wissinger B., Prokisch H., Stingl K., Weisschuh N. (2021). X-Linked Retinitis Pigmentosa Caused by Non-Canonical Splice Site Variants in *RPGR*. Int. J. Mol. Sci..

[29] Verbakel S.K., Fadaie Z., Klevering B.J., Van Genderen M.M., Feenstra I., Cremers F.P.M., Hoyng C.B., Roosing S. (2019). The identification of a RNA splice variant in TULP1 in two siblings with early-onset photoreceptor dystrophy. Mol. Genet. Genom. Med..

[30] Al-Khuzaei S., Broadgate S., Halford S., Jolly J.K., Shanks M., Clouston P., Downes S.M. (2020). Novel Pathogenic Sequence Variants in *NR2E3* and Clinical Findings in Three Patients. Genes.

[31] Chen Z., Moran K., Richards-Yutz J., Toorens E., Gerhart D., Ganguly T., Shields C.L., Ganguly A. (2014). Enhanced Sensitivity for Detection of Low-Level Germline MosaicRB1Mutations in Sporadic Retinoblastoma Cases Using Deep Semiconductor Sequencing. Hum. Mutat..

[32] Strubbe I., Van Cauwenbergh C., De Zaeytijd J., De Jaegere S., De Bruyne M., Rosseel T., Van de Sompele S., De Baere E., Leroy B.P. (2021). Phenocopy of a heterozygous carrier of X-linked retinitis pigmentosa due to mosaicism for a RHO variant. Sci. Rep..

[33] Dawod P.G.A., Jancic J., Marjanovic A., Brankovic M., Jankovic M., Samardzic J., Potkonjak D., Djuric V., Mesaros S., Novakovic I. (2020). Whole Mitochondrial Genome Analysis in Serbian Cases of Leber’s Hereditary Optic Neuropathy. Genes.

[34] Brusa R., Mauri E., Dell’Arti L., Magri F., Ronchi D., Minorini V., Mainetti C., Gagliardi D., Faravelli I., Meneri M. (2020). Expanding the clinical spectrum of the mitochondrial mutation A13084T in the ND5 gene. Neurol. Genet..

[35] Vincent A.L., Abeysekera N., Van Bysterveldt K.A., Oliver V.F., Ellingford J.M., Barton S., Black G.C. (2017). Next-generation sequencing targeted disease panel in rod-cone retinal dystrophies in Māori and Polynesian reveals novel changes and a common founder mutation. Clin. Exp. Ophthalmol..

[36] Numa S., Oishi A., Higasa K., Oishi M., Miyata M., Hasegawa T., Ikeda H.O., Otsuka Y., Matsuda F., Tsujikawa A. (2020). EYS is a major gene involved in retinitis pigmentosa in Japan: Genetic landscapes revealed by stepwise genetic screening. Sci. Rep..

[37] Sompele S.V.D., Smith C., Karali M., Corton M., Van Schil K., Peelman F., Cherry T., Rosseel T., Verdin H., Derolez J. (2018). Biallelic sequence and structural variants in RAX2 are a novel cause for autosomal recessive inherited retinal disease. Genet. Med..

[38] Glen W.B., Peterseim M.M.W., Badilla R., Znoyko I., Bourg A., Wilson R., Hardiman G., Wolff D., Martinez J. (2019). A high prevalence of biallelic RPE65 mutations in Costa Rican children with Leber congenital amaurosis and early-onset retinal dystrophy. Ophthalmic Genet..

[39] Avela K., Sankila E.-M., Seitsonen S., Kuuluvainen L., Barton S., Gillies S., Aittomäki K. (2018). A founder mutation inCERKLis a major cause of retinal dystrophy in Finland. Acta Ophthalmol..

[40] Avila-Fernandez A., Cortón M., Nishiguchi K.M., Muñoz-Sanz N., Benavides-Mori B., Blanco-Kelly F., Riveiro-Alvarez R., Garcia-Sandoval B., Rivolta C., Ayuso C. (2012). Identification of an RP1 Prevalent Founder Mutation and Related Phenotype in Spanish Patients with Early-Onset Autosomal Recessive Retinitis. Ophthalmology.

[41] Perea-Romero I., Gordo G., Iancu I.F., Del Pozo-Valero M., Almoguera B., Blanco-Kelly F., Carreño E., Jimenez-Rolando B., Lopez-Rodriguez R., Lorda-Sanchez I. (2021). Genetic landscape of 6089 inherited retinal dystrophies affected cases in Spain and their therapeutic and extended epidemiological implications. Sci. Rep..

[42] Tatour Y., Tamaiev J., Shamaly S., Colombo R., Bril E., Rabinowitz T., Yaakobi A., Mezer E., Leibu R., Tiosano B. (2019). A Novel Intronic Mutation of PDE6B Is a Major Cause of Autosomal Recessive Retinitis Pigmentosa among Caucasus Jews. Mol. Vis..

[43] Rehman A.U., Peter V.G., Quinodoz M., Rashid A., Khan S.A., Superti-Furga A., Rivolta C. (2019). Exploring the Genetic Landscape of Retinal Diseases in North-Western Pakistan Reveals a High Degree of Autozygosity and a Prevalent Founder Mutation in ABCA4. Genes.

[44] Reiner J., Pisani L., Qiao W., Singh R., Yang Y., Shi L., Khan W.A., Sebra R., Cohen N., Babu A. (2018). Cytogenomic identification and long-read single molecule real-time (SMRT) sequencing of a Bardet–Biedl Syndrome 9 (BBS9) deletion. NPJ Genom. Med..

[45] Ostergaard E., Duno M., Batbayli M., Vilhelmsen K., Rosenberg T. (2011). A Novel MERTK Deletion Is a Common Founder Muta-tion in the Faroe Islands and Is Responsible for a High Proportion of Retinitis Pigmentosa Cases. Mol. Vis..

[46] Thompson J.A., De Roach J.N., McLaren T.L., Montgomery H.E., Hoffmann L.H., Campbell I.R., Chen F.K., Mackey D.A., Lamey T.M. (2017). The genetic profile of Leber congenital amaurosis in an Australian cohort. Mol. Genet. Genom. Med..

[47] Motta F.L., Martin R.P., Filippelli-Silva R., Salles M.V., Sallum J.M.F. (2018). Relative frequency of inherited retinal dystrophies in Brazil. Sci. Rep..

[48] Liu X., Tao T., Zhao L., Li G., Yang L. (2021). Molecular diagnosis based on comprehensive genetic testing in 800 Chinese families with non-syndromic inherited retinal dystrophies. Clin. Exp. Ophthalmol..

[49] Gao F.-J., Li J.-K., Chen H., Hu F.-Y., Zhang S.-H., Qi Y.-H., Xu P., Wang D.-D., Wang L.-S., Chang Q. (2019). Genetic and Clinical Findings in a Large Cohort of Chinese Patients with Suspected Retinitis Pigmentosa. Ophthalmology.

[50] Liu X.Z., Li Y.Y., Yang L.P. (2020). Comparison study of whole exome sequencing and targeted panel sequencing in molecular diagnosis of inherited retinal dystrophies. Beijing Da Xue Xue Bao.

[51] Huang X.-F., Mao J.-Y., Huang Z.-Q., Rao F.-Q., Cheng F.-F., Li F.-F., Wang Q.-F., Jin Z.-B. (2017). Genome-Wide Detection of Copy Number Variations in Unsolved Inherited Retinal Disease. Investig. Ophthalmol. Vis. Sci..

[52] Dan H., Huang X., Xing Y., Shen Y. (2020). Application of targeted panel sequencing and whole exome sequencing for 76 Chinese families with retinitis pigmentosa. Mol. Genet. Genom. Med..

[53] Wang L., Zhang J., Chen N., Wang L., Zhang F., Ma Z., Li G., Yang L. (2018). Application of Whole Exome and Targeted Panel Sequencing in the Clinical Molecular Diagnosis of 319 Chinese Families with Inherited Retinal Dystrophy and Comparison Study. Genes.

[54] Avela K., Salonen-Kajander R., Laitinen A., Ramsden S., Barton S., Rudanko S. (2019). The genetic aetiology of retinal degeneration in children in Finland—New founder mutations identified. Acta Ophthalmol..

[55] Weisschuh N., Obermaier C.D., Battke F., Bernd A., Kuehlewein L., Nasser F., Zobor D., Zrenner E., Weber E., Wissinger B. (2020). Genetic architecture of inherited retinal degeneration in Germany: A large cohort study from a single diagnostic center over a 9-year period. Hum. Mutat..

[56] Birtel J., Eisenberger T., Gliem M., Müller P.L., Herrmann P., Betz C., Zahnleiter D., Neuhaus C., Lenzner S., Holz F.G. (2018). Clinical and genetic characteristics of 251 consecutive patients with macular and cone/cone-rod dystrophy. Sci. Rep..

[57] Tayebi N., Akinrinade O., Khan M.I., Hejazifar A., Dehghani A., Cremers F.P., Akhlaghi M. (2019). Targeted next generation sequencing reveals genetic defects underlying inherited retinal disease in Iranian families. Mol. Vis..

[58] Whelan L., Dockery A., Wynne N., Zhu J., Stephenson K., Silvestri G., Turner J., O’Byrne J.J., Carrigan M., Humphries P. (2020). Findings from a Genotyping Study of over 1000 People with Inherited Retinal Disorders in Ireland. Genes.

[59] Sharon D., Ben-Yosef T., Cohen N.G., Pras E., Gradstein L., Soudry S., Mezer E., Zur D., Abbasi A.H., Zeitz C. (2020). A nationwide genetic analysis of inherited retinal diseases in Israel as assessed by the Israeli inherited retinal disease consortium (IIRDC). Hum. Mutat..

[60] Koyanagi Y., Akiyama M., Nishiguchi K.M., Momozawa Y., Kamatani Y., Takata S., Inai C., Iwasaki Y., Kumano M., Murakami Y. (2019). Genetic characteristics of retinitis pigmentosa in 1204 Japanese patients. J. Med. Genet..

[61] Surl D., Shin S., Lee S.-T., Choi J.R., Lee J., Byeon S.H., Han S.-H., Lim H.T., Han J. (2020). Copy Number Variations and Multiallelic Variants in Korean Patients with Leber Congenital Amaurosis. Mol. Vis..

[62] Kim M.S., Joo K., Seong M.-W., Kim M.J., Park K.H., Park S.S., Woo S.J. (2019). Genetic Mutation Profiles in Korean Patients with Inherited Retinal Diseases. J. Korean Med. Sci..

[63] Zenteno J.C., García-Montaño L.A., Cruz-Aguilar M., Ronquillo J., Rodas-Serrano A., Aguilar-Castul L., Matsui R., Vencedor-Meraz C.I., Arce-González R., Graue-Wiechers F. (2019). Extensive genic and allelic heterogeneity underlying inherited retinal dystrophies in Mexican patients molecularly analyzed by next-generation sequencing. Mol. Genet. Genom. Med..

[64] Wawrocka A., Skorczyk-Werner A., Wicher K., Niedziela Z., Ploski R., Rydzanicz M., Sykulski M., Kociecki J., Weisschuh N., Kohl S. (2018). Novel variants identified with next-generation sequencing in Polish patients with cone-rod dystrophy. Mol. Vis..

[65] Martin-Merida I., Avila-Fernandez A., Del Pozo-Valero M., Blanco-Kelly F., Zurita O., Perez-Carro R., Aguilera-Garcia D., Riveiro-Alvarez R., Arteche A., Trujillo-Tiebas M.J. (2019). Genomic Landscape of Sporadic Retinitis Pigmentosa. Ophthalmology.

[66] Gonzàlez-Duarte R., De Castro-Miró M., Tuson M., Ramírez-Castañeda V., Gils R.V., Marfany G. (2019). Scaling New Heights in the Genetic Diagnosis of Inherited Retinal Dystrophies. Adv. Exp. Med. Biol..

[67] Diñeiro M., Capín R., Cifuentes G.Á., Fernández-Vega B., Villota E., Otero A., Santiago A., Pruneda P.C., Castillo D., Viejo-Díaz M. (2020). Comprehensive genomic diagnosis of inherited retinal and optical nerve disorders reveals hidden syndromes and personalized therapeutic options. Acta Ophthalmol..

[68] Chen Z.-J., Lin K.-H., Lee S.-H., Shen R.-J., Feng Z.-K., Wang X.-F., Ms X.H., Huang Z.-Q., Jin Z.-B. (2020). Mutation spectrum and genotype-phenotype correlation of inherited retinal dystrophy in Taiwan. Clin. Exp. Ophthalmol..

[69] Habibi I., Falfoul Y., Turki A., Hassairi A., El Matri K., Chebil A., Schorderet D.F., El Matri L. (2020). Genetic spectrum of retinal dystrophies in Tunisia. Sci. Rep..

[70] Khan A.O. (2019). Phenotype-guided genetic testing of pediatric inherited retinal disease in the United Arab Emirates. Retina.

[71] Patel N., Alkuraya H., Alzahrani S.S., Nowailaty S., Seidahmed M.Z., Alhemidan A., Ben-Omran T., Ghazi N., Al-Aqeel A., Al-Owain M. (2018). Mutations in known disease genes account for the majority of autosomal recessive retinal dystrophies. Clin. Genet..

[72] Jiman O.A., Taylor R.L., Lenassi E., Smith J.C., Douzgou S., Ellingford J.M., Barton S., Hardcastle C., Fletcher T., Campbell C. (2020). Diagnostic yield of panel-based genetic testing in syndromic inherited retinal disease. Eur. J. Hum. Genet..

[73] Shah M., Shanks M., Packham E., Williams J., Haysmoore J., MacLaren R.E., Németh A.H., Clouston P., Downes S.M. (2020). Next generation sequencing using phenotype-based panels for genetic testing in inherited retinal diseases. Ophthalmic Genet..

[74] Carss K.J., Arno G., Erwood M., Stephens J., Sanchis-Juan A., Hull S., Megy K., Grozeva D., Dewhurst E., Malka S. (2017). Comprehensive rare variant analysis via whole-genome sequencing to determine the molecular pathology of inherited retinal disease. Am. J. Hum. Genet..

[75] Lenassi E., Clayton-Smith J., Douzgou S., Ramsden S.C., Ingram S., Hall G., Hardcastle C.L., Fletcher T.A., Taylor R.L., Ellingford J.M. (2020). Clinical utility of genetic testing in 201 preschool children with inherited eye disorders. Genet. Med..

[76] Patel A., Hayward J.D., Tailor V., Nyanhete R., Ahlfors H., Gabriel C., Jannini T.B., Abbou-Rayyah Y., Henderson R., Nischal K.K. (2019). The Oculome Panel Test. Ophthalmology.

[77] Taylor R.L., Parry N.R., Barton S.J., Campbell C., Delaney C.M., Ellingford J.M., Hall G., Hardcastle C., Morarji J., Nichol E.J. (2017). Panel-Based Clinical Genetic Testing in 85 Children with Inherited Retinal Disease. Ophthalmology.

[78] Goetz K.E., Reeves M.J., Gagadam S., Blain D., Bender C., Lwin C., Naik A., Tumminia S.J., Hufnagel R.B. (2020). Genetic testing for inherited eye conditions in over 6,000 individuals through the eyeGENE network. Am. J. Med. Genet. Part C Semin. Med. Genet..

[79] Stone E.M., Andorf J.L., Whitmore S.S., DeLuca A.P., Giacalone J.C., Streb L.M., Braun T.A., Mullins R.F., Scheetz T.E., Sheffield V.C. (2017). Clinically Focused Molecular Investigation of 1000 Consecutive Families with Inherited Retinal Disease. Ophthalmology.

[80] Bryant L., Lozynska O., Maguire A.M., Aleman T.S., Bennett J. (2017). Prescreening whole exome sequencing results from patients with retinal degeneration for variants in genes associated with retinal degeneration. Clin. Ophthalmol..

[81] Dillon O.J., Lunke S., Stark Z., Yeung A., Thorne N., Gaff C., White S.M., Tan T.Y. (2018). Exome sequencing has higher diagnostic yield compared to simulated disease-specific panels in children with suspected monogenic disorders. Eur. J. Hum. Genet..

[82] Pontikos N., Arno G., Jurkute N., Schiff E., Ba-Abbad R., Malka S., Gimenez A., Georgiou M., Wright G., Armengol M. (2020). Genetic Basis of Inherited Retinal Disease in a Molecularly Characterized Cohort of More Than 3000 Families from the United Kingdom. Ophthalmology.

[83] Hanany M., Sharon D. (2019). Allele frequency analysis of variants reported to cause autosomal dominant inherited retinal diseases question the involvement of 19% of genes and 10% of reported pathogenic variants. J. Med. Genet..

[84] Dockery A., Carrigan M., Wynne N., Stephenson K., Keegan D., Kenna P.F., Farrar G.J. (2019). A Novel FLVCR1 Variant Implicated in Retinitis Pigmentosa. Adv. Exp. Med. Biol..

[85] Jones K.D., Wheaton D.K., Bowne S.J., Sullivan L.S., Birch D.G., Chen R., Daiger S.P. (2017). Next-generation sequencing to solve complex inherited retinal dystrophy: A case series of multiple genes contributing to disease in extended families. Mol. Vis..

[86] Birtel J., Gliem M., Hess K., Birtel T.H., Holz F.G., Zechner U., Bolz H.J., Herrmann P. (2020). Comprehensive Geno- and Phenotyping in a Complex Pedigree Including Four Different Inherited Retinal Dystrophies. Genes.

[87] Rodríguez-Muñoz A., García-Bohórquez B., Udaondo P., Hervás-Ontiveros A., Salom D., Aller E., Jaijo T., García-García G., Millán J.M. (2021). Concomitant mutations in inherited retinal dystrophies. Retina.

[88] Lelieveld S.H., Spielmann M., Mundlos S., Veltman J., Gilissen C. (2015). Comparison of Exome and Genome Sequencing Technologies for the Complete Capture of Protein-Coding Regions. Hum. Mutat..

[89] Schwarze K., Buchanan J., Taylor J.C., Wordsworth S. (2018). Are whole-exome and whole-genome sequencing approaches cost-effective? A systematic review of the literature. Genet. Med..

[90] MassGenomics Brace Yourself for Large-Scale Whole Genome Sequencing. http://massgenomics.org/2014/11/brace-yourself-for-large-scale-whole-genome-sequencing.html.

[91] Approximate Sizes of Sequencing Run Output Folders. https://support.illumina.com/bulletins/2018/01/approximate-sizes-of-sequencing-run-output-folders.html.

[92] Strand NGS Storage and Computation Requirements. https://www.strand-ngs.com/support/ngs-data-storage-requirements.

[93] CRAM Benchmarks. http://www.htslib.org/benchmarks/CRAM.html.

[94] Krumm N., Hoffman N. (2020). Practical estimation of cloud storage costs for clinical genomic data. Pract. Lab. Med..

[95] Tanjo T., Kawai Y., Tokunaga K., Ogasawara O., Nagasaki M. (2021). Practical guide for managing large-scale human genome data in research. J. Hum. Genet..

[96] Hart M.R., Biesecker B.B., Blout C.L., Christensen K.D., Amendola L.M., Bergstrom K.L., Biswas S., Bowling K.M., Brothers K.B., Conlin L.K. (2019). Secondary findings from clinical genomic sequencing: Prevalence, patient perspectives, family history assessment, and health-care costs from a multisite study. Genet. Med..

[97] Green R.C., Berg J.S., Grody W.W., Kalia S.S., Korf B.R., Martin C.L., JD A.L.M., Nussbaum R.L., O’Daniel J.M., Ormond K.E. (2013). ACMG recommendations for reporting of incidental findings in clinical exome and genome sequencing. Genet. Med..

[98] Kalia S.S., Adelman K., Bale S.J., Chung W.K., Eng C., Evans J.P., Herman G.E., Hufnagel S.B., Klein T.E., Korf B.R. (2017). Recommendations for reporting of secondary findings in clinical exome and genome sequencing, 2016 update (ACMG SF v2.0): A policy statement of the American College of Medical Genetics and Genomics. Genet. Med..

[99] Miller D.T., Lee K., Gordon A.S., Amendola L.M., Adelman K., Bale S.J., Chung W.K., Gollob M.H., Harrison S.M., Herman G.E. (2021). Recommendations for reporting of secondary findings in clinical exome and genome sequencing, 2021 update: A policy statement of the American College of Medical Genetics and Genomics (ACMG). Genet. Med..

[100] Miller D.T., Lee K., Chung W.K., Gordon A.S., Herman G.E., Klein T.E., Stewart D.R., Amendola L.M., Adelman K., Bale S.J. (2021). ACMG SF v3.0 list for reporting of secondary findings in clinical exome and genome sequencing: A policy statement of the American College of Medical Genetics and Genomics (ACMG). Genet. Med..

[101] Lionel A.C., Costain G., Monfared N., Walker S., Reuter M.S., Hosseini S.M., Thiruvahindrapuram B., Merico D., Jobling R., Nalpathamkalam T. (2018). Improved diagnostic yield compared with targeted gene sequencing panels suggests a role for whole-genome sequencing as a first-tier genetic test. Genet. Med..

[102] Zeitz C., Michiels C., Neuillé M., Friedburg C., Condroyer C., Boyard F., Antonio A., Bouzidi N., Milicevic D., Veaux R. (2019). Where are the missing gene defects in inherited retinal disorders? Intronic and synonymous variants contribute at least to 4% of CACNA1F -mediated inherited retinal disorders. Hum. Mutat..

[103] Khan M., Cornelis S.S., Pozo-Valero M.D., Whelan L., Runhart E.H., Mishra K., Bults F., AlSwaiti Y., AlTalbishi A., De Baere E. (2020). Resolving the dark matter of ABCA4 for 1054 Stargardt disease probands through integrated genomics and transcriptomics. Genet. Med..

[104] Almomani R., Marchi M., Sopacua M., Lindsey P., Salvi E., De Koning B., Santoro S., Magri S., Smeets H.J.M., Boneschi F.M. (2020). Evaluation of molecular inversion probe versus TruSeq^®^ custom methods for targeted next-generation sequencing. PLoS ONE.

[105] Pozo M.G.-D., Martín-Sánchez M., Bravo-Gil N., Méndez-Vidal C., Chimenea Á., La Rúa E.R.-D., Borrego S., Antiñolo G. (2018). Searching the second hit in patients with inherited retinal dystrophies and monoallelic variants in ABCA4, USH2A and CEP290 by whole-gene targeted sequencing. Sci. Rep..

[106] Vervoort R., Lennon A., Bird A.C., Tulloch B., Axton R., Miano M.G., Meindl A., Meitinger T., Ciccodicola A., Wright A.F. (2000). Mutational hot spot within a new RPGR exon in X-linked retinitis pigmentosa. Nat. Genet..

[107] Di Iorio V., Karali M., Melillo P., Testa F., Brunetti-Pierri R., Musacchia F., Condroyer C., Neidhardt J., Audo I., Zeitz C. (2020). Spectrum of Disease Severity in Patients with X-Linked Retinitis Pigmentosa Due to RPGR Mutations. Investig. Ophthalmol. Vis. Sci..

[108] Chiang J.P.W., Lamey T.M., Wang N.K., Duan J., Zhou W., McLaren T.L., Thompson J.A., Ruddle J., De Roach J.N. (2018). Development of High-Throughput Clinical Testing ofRPGRORF15 Using a Large Inherited Retinal Dystrophy Cohort. Investig. Ophthalmol. Vis. Sci..

[109] Maggi J., Roberts L., Koller S., Rebello G., Berger W., Ramesar R. (2020). De Novo Assembly-Based Analysis of *RPGR* Exon ORF15 in an Indigenous African Cohort Overcomes Limitations of a Standard Next-Generation Sequencing (NGS) Data Analysis Pipeline. Genes.

[110] Deeb S. (2005). The molecular basis of variation in human color vision. Clin. Genet..

[111] Atilano S.R., Kenney M.C., Briscoe A.D., Jameson K.A. (2020). A two-step method for identifying photopigment opsin and rhodopsin gene sequences underlying human color vision phenotypes. Mol. Vis..

[112] Ueyama H., Li Y.-H., Fu G.-L., Lertrit P., Atchaneeyasakul L.-O., Oda S., Tanabe S., Nishida Y., Yamade S., Ohkubo I. (2003). An A-71C substitution in a green gene at the second position in the red/green visual-pigment gene array is associated with deutan color-vision deficiency. Proc. Natl. Acad. Sci. USA.

[113] Katagiri S., Iwasa M., Hayashi T., Hosono K., Yamashita T., Kuniyoshi K., Ueno S., Kondo M., Ueyama H., Ogita H. (2018). Genotype determination of the OPN1LW/OPN1MW genes: Novel disease-causing mechanisms in Japanese patients h blue cone monochromacy. Sci. Rep..

[114] Yatsenko S., Bakos H., Vitullo K., Kedrov M., Kishore A., Jennings B., Surti U., Wood-Trageser M., Cercone S., Yatsenko A. (2015). High-resolution microarray analysis unravels complex Xq28 aberrations in patients and carriers affected by X-linked blue cone monochromacy. Clin. Genet..

[115] Wang C., Hosono K., Kachi S., Suto K., Nakamura M., Terasaki H., Miyake Y., Hotta Y., Minoshima S. (2016). Novel OPN1LW/OPN1MW deletion mutations in 2 Japanese families with blue cone monochromacy. Hum. Genome Var..

[116] Radziwon A., Arno G., Wheaton D.K., McDonagh E.M., Baple E.L., Webb-Jones K., Birch D.G., Webster A.R., Macdonald I.M. (2017). Single-base substitutions in the CHM promoter as a cause of choroideremia. Hum. Mutat..

[117] Coppieters F., Todeschini A.L., Fujimaki T., Baert A., De Bruyne M., Van Cauwenbergh C., Verdin H., Bauwens M., Ongenaert M., Kondo M. (2015). Hidden Genetic Variation in LCA9-Associated Congenital Blindness Explained by 5′UTR Mutations and Copy-Number Variations of NMNAT1. Hum. Mutat..

[118] Donato L., Scimone C., Rinaldi C., Aragona P., Briuglia S., D’Ascola A., D’Angelo R., Sidoti A. (2018). Stargardt Phenotype Associated with Two ELOVL4 Promoter Variants and ELOVL4 Downregulation: New Possible Perspective to Etiopathogenesis?. Investig. Ophthalmol. Vis. Sci..

[119] Van Schil K., Naessens S., Van De Sompele S., Carron M., Aslanidis A., Van Cauwenbergh C., Mayer A.K., Van Heetvelde M., Bauwens M., Verdin H. (2017). Mapping the genomic landscape of inherited retinal disease genes prioritizes genes prone to coding and noncoding copy-number variations. Genet. Med..

[120] Richards S., Aziz N., Bale S., Bick D., Das S., Gastier-Foster J., Grody W.W., Hegde M., Lyon E., Spector E. (2015). Standards and guidelines for the interpretation of sequence variants: A joint consensus recommendation of the American College of Medical Genetics and Genomics and the Association for Molecular Pathology. Genet. Med..

[121] Brandt T., Sack L.M., Arjona D., Tan D., Mei H., Cui H., Gao H., Bean L.J.H., Ankala A., Del Gaudio D. (2019). Adapting ACMG/AMP sequence variant classification guidelines for single-gene copy-number variants. Genet. Med..

[122] Li H.-P., Yuan S.-Q., Wang X.-G., Sheng X.-L., Li X.-R. (2020). Myopia with X-linked retinitis pigmentosa results from a novel gross deletion of RPGR gene. Int. J. Ophthalmol..

[123] Buena-Atienza E., Nasser F., Kohl S., Wissinger B. (2018). A 73,128 bp de novo deletion encompassing the OPN1LW/OPN1MW gene cluster in sporadic Blue Cone Monochromacy: A case report. BMC Med. Genet..

[124] De Bruijn S.E., Fiorentino A., Ottaviani D., Fanucchi S., Melo U.S., Corral-Serrano J.C., Mulders T., Georgiou M., Rivolta C., Pontikos N. (2020). Structural Variants Create New Topological-Associated Domains and Ectopic Retinal Enhancer-Gene Contact in Dominant Retinitis Pigmentosa. Am. J. Hum. Genet..

[125] Jones K.D., Radziwon A., Birch D.G., MacDonald I.M. (2020). A novel SVA retrotransposon insertion in the CHM gene results in loss of REP-1 causing choroideremia. Ophthalmic Genet..

[126] Hurk J.A.J.M.V.D., Van De Pol D.J.R., Wissinger B., Van Driel M.A., Hoefsloot L.H., De Wijs I.J., Born L.I.V.D., Heckenlively J.R., Brunner H.G., Zrenner E. (2003). Novel types of mutation in the choroideremia (CHM) gene: A full-length L1 insertion and an intronic mutation activating a cryptic exon. Qual. Life Res..

[127] Hitti-Malin R.J., Burmeister L.M., Ricketts S.L., Lewis T.W., Pettitt L., Boursnell M., Schofield E.C., Sargan D., Mellersh C.S. (2020). A LINE-1 insertion situated in the promoter of IMPG2 is associated with autosomal recessive progressive retinal atrophy in Lhasa Apso dogs. BMC Genet..

[128] Tavares E., Tang C.Y., Vig A., Li S., Billingsley G., Sung W., Vincent A., Thiruvahindrapuram B., Héon E. (2018). Retrotransposon insertion as a novel mutational event in Bardet-Biedl syndrome. Mol. Genet. Genom. Med..

[129] Delvallée C., Nicaise S., Antin M., Leuvrey A.-S., Nourisson E., Leitch C.C., Kellaris G., Stoetzel C., Geoffroy V., Scheidecker S. (2020). A BBS1 SVA F retrotransposon insertion is a frequent cause of Bardet-Biedl syndrome. Clin. Genet..

[130] Ma X., Fan J., Wu Y., Zhao S., Zheng X., Sun C., Tan L. (2020). Whole-genome de novo assemblies reveal extensive structural variations and dynamic organelle-to-nucleus DNA transfers in African and Asian rice. Plant J..

[131] Zampaglione E., Kinde B., Place E.M., Navarro-Gomez D., Ma C.F., Jamshidi F., Nassiri S., Mazzone J.A., Finn C., Schlegel D. (2020). Copy-number variation contributes 9% of pathogenicity in the inherited retinal degenerations. Genet. Med..

[132] Bujakowska K.M., Fernandez-Godino R., Place E., Consugar M., Navarro-Gomez D., White J., Bedoukian E.C., Zhu X., Xie H.M., Gai X. (2016). Copy-number variation is an important contributor to the genetic causality of inherited retinal degenerations. Genet. Med..

[133] Ellingford J.M., Horn B., Campbell C., Arno G., Barton S., Tate C., Bhaskar S., Sergouniotis P.I., Taylor R.L., Carss K.J. (2017). Assessment of the incorporation of CNV surveillance into gene panel next-generation sequencing testing for inherited retinal diseases. J. Med. Genet..

[134] Dong X., Liu B., Yang L., Wang H., Wu B., Liu R., Chen H., Chen X., Yu S., Chen B. (2020). Clinical exome sequencing as the first-tier test for diagnosing developmental disorders covering both CNV and SNV: A Chinese cohort. J. Med. Genet..

[135] Sloan-Heggen C.M., Bierer A.O., Shearer A.E., Kolbe D.L., Nishimura C.J., Frees K.L., Ephraim S.S., Shibata S.B., Booth K.T., Campbell C. (2016). Comprehensive genetic testing in the clinical evaluation of 1119 patients with hearing loss. Qual. Life Res..

[136] Sanchez-Navarro I., Da Silva L.R.J., Blanco-Kelly F., Zurita O., Sanchez-Bolivar N., Villaverde C., Lopez-Molina M.I., Garcia-Sandoval B., Swafiri S.T.S., Minguez P. (2018). Combining targeted panel-based resequencing and copy-number variation analysis for the diagnosis of inherited syndromic retinopathies and associated ciliopathies. Sci. Rep..

[137] Goodwin S., McPherson J.D., McCombie W.R. (2016). Coming of age: Ten years of next-generation sequencing technologies. Nat. Rev. Genet..

[138] De Koning A.P.J., Gu W., Castoe T.A., Batzer M.A., Pollock D.D. (2011). Repetitive Elements May Comprise Over Two-Thirds of the Human Genome. PLoS Genet..

[139] Chaisson M.J.P., Wilson R.K., Eichler E.E. (2015). Genetic variation and the de novo assembly of human genomes. Nat. Rev. Genet..

[140] Mantere T., Kersten S., Hoischen A. (2019). Long-Read Sequencing Emerging in Medical Genetics. Front. Genet..

[141] Botton M.R., Yang Y., Scott E.R., Desnick R.J., Scott S.A. (2020). Phased Haplotype Resolution of the *SLC6A4* Promoter Using Long-Read Single Molecule Real-Time (SMRT) Sequencing. Genes.

[142] Sanchis-Juan A., Stephens J., French C.E., Gleadall N., Mégy K., Penkett C., Shamardina O., Stirrups K., Delon I., Dewhurst E. (2018). Complex structural variants in Mendelian disorders: Identification and breakpoint resolution using short- and long-read genome sequencing. Genome Med..

[143] Beales P.L., Badano J.L., Ross A.J., Ansley S.J., Hoskins B.E., Kirsten B., Mein C.A., Froguel P., Scambler P.J., Lewis R.A. (2003). Genetic Interaction of BBS1 Mutations with Alleles at Other BBS Loci Can Result in Non-Mendelian Bardet-Biedl Syndrome. Am. J. Hum. Genet..

[144] Wheway G., Douglas A., Baralle D., Guillot E. (2020). Mutation spectrum of PRPF31, genotype-phenotype correlation in retinitis pigmentosa, and opportunities for therapy. Exp. Eye Res..

[145] Pormehr L.A., Ahmadian S., Daftarian N., Mousavi S.A., Shafiezadeh M. (2019). PRPF31 reduction causes mis-splicing of the phototransduction genes in human organotypic retinal culture. Eur. J. Hum. Genet..

[146] Yang D., Yao Q., Li Y., Xu Y., Wang J., Zhao H., Liu F., Zhang Z., Liu Y., Bie X. (2020). A c.544_618del75bp mutation in the splicing factor gene PRPF31 is involved in non-syndromic retinitis pigmentosa by reducing the level of mRNA expression. Ophthalmic Physiol. Opt..

[147] Rose A.M., Shah A.Z., Venturini G., Krishna A., Chakravarti A., Rivolta C., Bhattacharya S.S. (2016). Transcriptional regulation of PRPF31 gene expression by MSR1 repeat elements causes incomplete penetrance in retinitis pigmentosa. Sci. Rep..

[148] Green D.J., Sallah S.R., Ellingford J.M., Lovell S.C., Sergouniotis P.I. (2020). Variability in Gene Expression is Associated with Incomplete Penetrance in Inherited Eye Disorders. Genes.

[149] The Genome Aggregation Database. https://gnomad.broadinstitute.org/.

[150] Sangermano R., Khan M., Cornelis S.S., Richelle V., Albert S., Garanto A., Elmelik D., Qamar R., Lugtenberg D., Born L.I.V.D. (2017). ABCA4 midigenes reveal the full splice spectrum of all reported noncanonical splice site variants in Stargardt disease. Genome Res..

[151] Di Scipio M., Tavares E., Deshmukh S., Audo I., Green-Sanderson K., Zubak Y., Zine-Eddine F., Pearson A., Vig A., Tang C.Y. (2020). Phenotype Driven Analysis of Whole Genome Sequencing Identifies Deep Intronic Variants that Cause Retinal Dystrophies by Aberrant Exonization. Investig. Ophthalmol. Vis. Sci..

[152] Fadaie Z., Khan M., Del Pozo-Valero M., Cornelis S.S., Ayuso C., Cremers F.P.M., Roosing S. (2019). Identification of splice defects due to noncanonical splice site or deep-intronic variants in ABCA4. Hum. Mutat..

[153] Jonsson F., Westin I.M., Österman L., Sandgren O., Burstedt M., Holmberg M., Golovleva I. (2018). ATP-binding cassette subfamily A, member 4 intronic variants c.4773+3A>G and c.5461-10T>C cause Stargardt disease due to defective splicing. Acta Ophthalmol..

[154] Vig A., Poulter J.A., Poulter J.A., Ottaviani D., Tavares E., Toropova K., Tracewska A.M., Mollica A., Kang J., Kehelwathugoda O. (2020). DYNC2H1 hypomorphic or retina-predominant variants cause nonsyndromic retinal degeneration. Genet. Med..

[155] Brooks M.J., Chen H.Y., Kelley R.A., Mondal A.K., Nagashima K., De Val N., Li T., Chaitankar V., Swaroop A. (2019). Improved Retinal Organoid Differentiation by Modulating Signaling Pathways Revealed by Comparative Transcriptome Analyses with Development In Vivo. Stem Cell Rep..

[156] Cowan A.S., Renner M., De Gennaro M., Roma G., Nigsch F., Roska B., Cowan C.S., Gross-Scherf B., Goldblum D., Hou Y. (2020). Cell Types of the Human Retina and Its Organoids at Single-Cell Resolution. Cell.

[157] Fahim A.T., Sullivan L.S., Bowne S.J., Jones K.D., Wheaton D.K., Khan N.W., Heckenlively J.R., Jayasundera K.T., Branham K.H., Andrews C.A. (2020). X-Chromosome Inactivation Is a Biomarker of Clinical Severity in Female Carriers of RPGR-Associated X-Linked Retinitis Pigmentosa. Ophthalmol. Retin..

[158] Zhang Q., Giacalone J.C., Searby C., Stone E.M., Tucker B.A., Sheffield V.C. (2019). Disruption of RPGR protein interaction network is the common feature of RPGR missense variations that cause XLRP. Proc. Natl. Acad. Sci. USA.

[159] Fahim A.T., Bowne S.J., Sullivan L.S., Webb K.D., Williams J.T., Wheaton D.K., Birch D.G., Daiger S.P. (2011). Polymorphic variation of RPGRIP1L and IQCB1 as modifiers of X-linked retinitis pigmentosa caused by mutations in RPGR. Single Mol. Single Cell Seq..

[160] Li Y., Xia X., Paulus Y.M. (2018). Advances in Retinal Optical Imaging. Photonics.

[161] Litts K.M., Woertz E.N., Georgiou M., Patterson E.J., Lam B.L., Fishman G.A., Pennesi M.E., Kay C.N., Hauswirth W.W., Michaelides M. (2021). Optical Coherence Tomography Artifacts Are Associated with Adaptive Optics Scanning Light Ophthalmoscopy Success in Achromatopsia. Transl. Vis. Sci. Technol..

[162] Russakoff D.B., Lamin A., Oakley J.D., Dubis A.M., Sivaprasad S. (2019). Deep Learning for Prediction of AMD Progression: A Pilot Study. Investig. Ophthalmol. Vis. Sci..

[163] Schmidt-Erfurth U., Waldstein S.M., Klimscha S., Sadeghipour A., Hu X., Gerendas B.S., Osborne A., Bogunovic H. (2018). Prediction of Individual Disease Conversion in Early AMD Using Artificial Intelligence. Investig. Ophthalmol. Vis. Sci..

[164] Schmidt-Erfurth U., Bogunovic H., Sadeghipour A., Schlegl T., Langs G., Gerendas B.S., Osborne A., Waldstein S.M. (2018). Machine Learning to Analyze the Prognostic Value of Current Imaging Biomarkers in Neovascular Age-Related Macular Degeneration. Ophthalmol. Retin..

[165] Burlina P.M., Joshi N., Pacheco K.D., Freund D.E., Kong J., Bressler N.M. (2018). Use of Deep Learning for Detailed Severity Characterization and Estimation of 5-Year Risk Among Patients with Age-Related Macular Degeneration. JAMA Ophthalmol..

[166] Burlina P., Joshi N., Pacheco K.D., Freund D.E., Kong J., Bressler N.M. (2018). Utility of Deep Learning Methods for Referability Classification of Age-Related Macular Degeneration. JAMA Ophthalmol..

[167] Grassmann F., Mengelkamp J., Brandl C., Harsch S., Zimmermann M.E., Linkohr B., Peters A., Heid I.M., Palm C., Weber B.H. (2018). A Deep Learning Algorithm for Prediction of Age-Related Eye Disease Study Severity Scale for Age-Related Macular Degeneration from Color Fundus Photography. Ophthalmology.

[168] Goldhagen B.E., Al-Khersan H. (2020). Diving Deep into Deep Learning: An Update on Artificial Intelligence in Retina. Curr. Ophthalmol. Rep..

[169] Peng Y., Dharssi S., Chen Q., Keenan T.D., Agrón E., Wong W.T., Chew E.Y., Lu Z. (2019). DeepSeeNet: A Deep Learning Model for Automated Classification of Patient-based Age-related Macular Degeneration Severity from Color Fundus Photographs. Ophthalmology.

[170] Charng J., Xiao D., Mehdizadeh M., Attia M.S., Arunachalam S., Lamey T.M., Thompson J.A., McLaren T.L., De Roach J.N., Mackey D.A. (2020). Deep learning segmentation of hyperautofluorescent fleck lesions in Stargardt disease. Sci. Rep..

[171] Ha A., Sun S., Kim Y.K., Lee J., Jeoung J.W., Kim H.C., Park K.H. (2020). Deep-learning-based enhanced optic-disc photography. PLoS ONE.

[172] Galvin O., Chi G., Brady L., Hippert C., Rubido M.D.V., Daly A., Michaelides M. (2020). The Impact of Inherited Retinal Diseases in the Republic of Ireland (ROI) and the United Kingdom (UK) from a Cost-of-Illness Perspective. Clin. Ophthalmol..

[173] McVeigh E., Jones H., Black G., Hall G. (2019). The psychosocial and service delivery impact of genomic testing for inherited retinal dystrophies. J. Community Genet..

[174] Sabbaghi H., Daftarian N., Suri F., Mirrahimi M., Madani S., Sheikhtaheri A., Khorrami F., Saviz P., Nejad M.Z., Tivay A. (2020). The First Inherited Retinal Disease Registry in Iran: Research Protocol and Results of a Pilot Study. Arch. Iran. Med..

[175] Marques J.P., Carvalho A.L., Henriques J., Murta J.N., Saraiva J., Silva R. (2020). Design, development and deployment of a web-based interoperable registry for inherited retinal dystrophies in Portugal: The IRD-PT. Orphanet J. Rare Dis..

[176] Lejeune C., Amado I.F. (2021). Valuing genetic and genomic testing in France: Current challenges and latest evidence. J. Community Genet..

[177] Sergouniotis P.I. (2019). Inherited Retinal Disorders: Using Evidence as a Driver for Implementation. Ophthalmology.

